# Response: Commentary: Low-dose ionizing radiation and the exposure–lag response: protocol for a prospective cohort study on The Health Effects of Chongqing Occupational Radiation Workers

**DOI:** 10.3389/fpubh.2026.1790438

**Published:** 2026-05-18

**Authors:** Qiang Huang, Xiao-Ling Qin, Wei Li

**Affiliations:** 1 Chongqing Center for Disease Control and Prevention, Chongqing, China; 2 School of Public Health, Chongqing Medical University, Chongqing, China

**Keywords:** cohort, distributed lag non-linear model, exposure-lag response, low-dose ionizing radiation, radiation workers

We sincerely appreciate Colin R. Muirhead's thoughtful comments on our protocol titled “Low-dose ionizing radiation and the exposure-lag response: protocol for a prospective cohort study on The Health Effects of Chongqing Occupational Radiation Workers.” Your in-depth insights into epidemiological study design provided invaluable guidance for strengthening our research protocol. In the following sections, we will address each of your concerns in detail.

Firstly, we prepared our study protocol in accordance with the STROBE Statement guidelines. The completed STROBE checklist, along with the relevant SPIROS checklist items, is provided as an attachment for reference.

Secondly, control of noise as a potential confounding factor, the necessary information has now been included. Specifically, in Section 2.6 (Baseline Information), we state that each worker's full occupational history will be retrieved from the Chongqing CDC Personal Dose Monitoring System, and that the baseline questionnaire explicitly records “contact history of toxic and hazardous substances.” This item specifically captures noise exposure, which will be quantitatively coded—including years of exposure and, where available, peak decibel levels—and used as a covariate in all relevant statistical models.

Thirdly, in estimating the sample size for assessing the exposure-lag relationship, we would ideally base our calculations on a study utilizing the Distributed Lag Non-Linear Model (DLNM) to analyze changes in blood cell counts under radiation exposure. However, to date, no studies have employed the DLNM model to examine the association between radiation exposure and blood cell changes in radiation workers. As such, we aim to detect a minimum difference of 0.18 between two groups to estimate an appropriate sample size. Following the application of the DLNM to identify potential exposure-lag responses, we will perform a *post-hoc* power analysis to ensure that the study achieves statistical power exceeding 90%.

The sample size was determined based on our primary outcome (1), which is the complete blood count. For studies that involve multiple endpoint events, the primary outcome is critical in sample size determination (2). Primary outcome measures must be sensitive to changes in the target population (2). Bone marrow is the most sensitive to ionizing radiation, and the peripheral blood cell count changes relatively early when the exposed dose exceeds the human tolerance level (3, 4). Complete blood count includes multiple indicators, such as white blood cells, red blood cells, and platelets. Two meta-analyses demonstrated that white blood cells exhibit abnormal changes at an early stage in response to low-dose ionizing radiation exposure (5, 6). Therefore, white blood cells were selected as the reference indicator for sample size calculation in this study.

**Figure 1:**
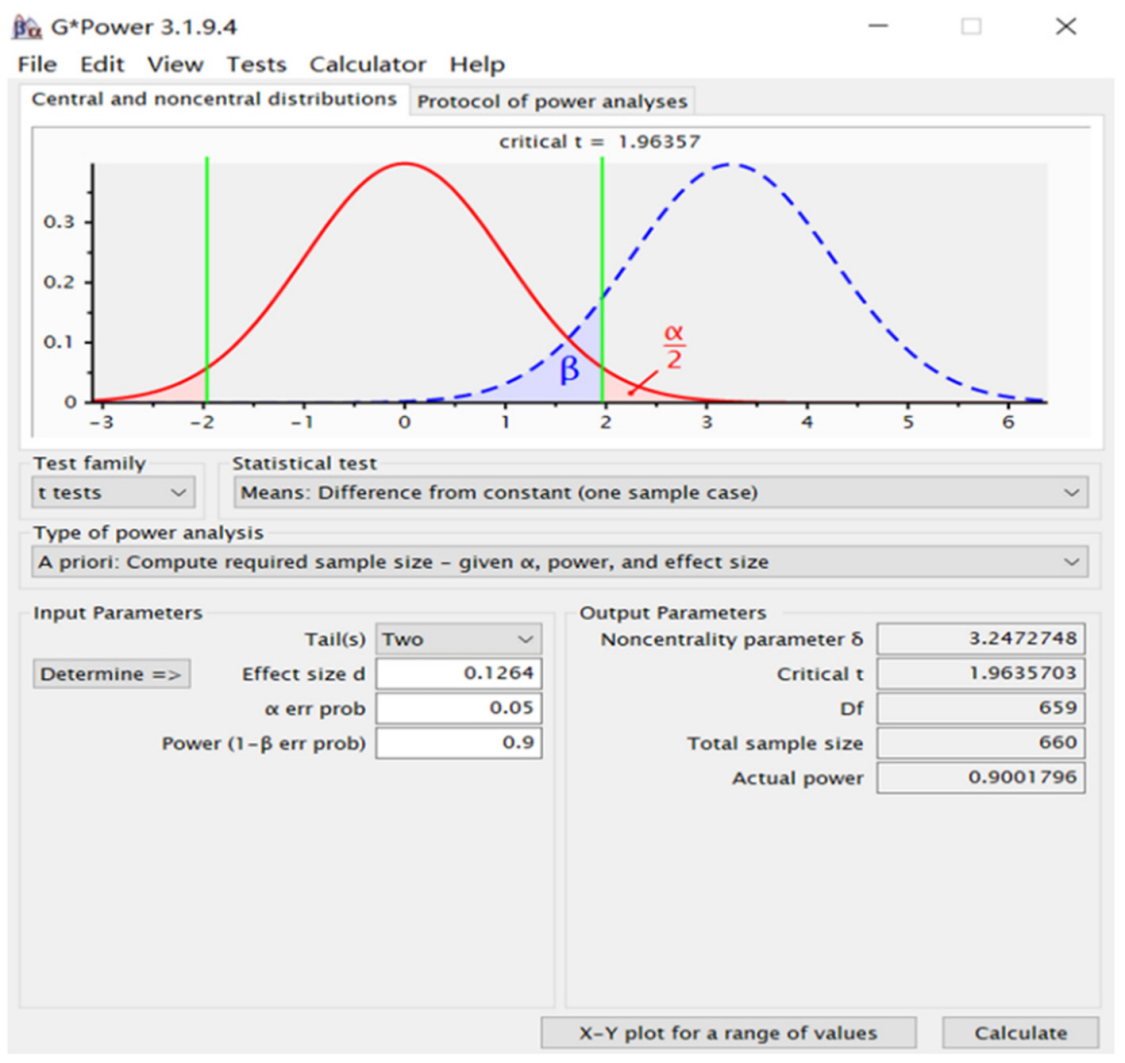
The screenshot of sample size calculation using G*Power.

Sample size calculation was performed using GPower software. The screenshot illustrating the use of G^*^Power for sample size calculation is presented in Figure 1.

We calculate the sample size based on the primary outcome (white blood cells). The sample size is calculated using this equation as follows:


$$\begin{array}{l} n={\left[\frac{\left(t_{\frac{\alpha }{2}}+t_{\beta }\right)\sigma }{\delta }\right]}^{2} \end{array}$$

*n* = the sample size, *n*=660;

*t*_α/2_= 1.96, for a significance level of 5%;

*t*_β_= 1.28, statistical power of 90%;

σ = the population standard deviation, according to the reference, σ = 1.39 (7);

δ = the minimum difference between the sample mean and the population mean that we want to detect, according to the reference, δ = 0.18 (7);

The loss follow-up rate is expected to be 20%.

Therefore, the final sample size is 825.

Finally, Large-scale cohort studies such as the International Nuclear Workers Cohort Study (INWORKS) (8) provide solid epidemiological evidence for radiation protection but which are designed to accurately quantify the carcinogenic risk of low-dose radiation, requiring a large sample size to detect rare events. However, this study focused on early biomarker changes, such as white blood cell count, thyroid hormone, skin damage, rather than cancer mortality, biomarkers have higher sensitivity and shorter latency (9).

We again express our gratitude to Colin R. Muirhead for their time, expertise, and dedication to advancing high-quality epidemiological research.
